# Quality assessment of systematic reviews regarding the effectiveness of zygomatic implants: an overview of systematic reviews

**DOI:** 10.4317/medoral.23569

**Published:** 2020-06-10

**Authors:** Pedro Henrique da Hora Sales, Marcus Vinícius Silva Weigel Gomes, Olavo Barbosa de Oliveira-Neto, Fernando José Camello de Lima, Jair Carneiro Leão

**Affiliations:** 1 DDS, MSc student, Department of Prosthesis and Oral and Maxillofacial Surgery, Dental School, Federal University of Pernambuco, Recife, Brazil; 2 Undergraduate student, Dental School, Federal University of Alagoas, Maceió, Brazil; 3 DDS, MSc, PhD Student, Institute of Health and Biological Sciences, Federal University of Alagoas, Maceió, Brazil; 4 DDS, MSc, PhD. Adjunct Professor, Human Anatomy Area, Institute of Health and Biological Sciences, Federal University of Alagoas, Maceió, Brazil; 5 DDS, MSc, PhD, Full Professor, Department of Clinical and Preventive Dentistry, Dental School, Federal University of Pernambuco, Recife, Brazil

## Abstract

**Background:**

Oral rehabilitation of atrophic maxillae features high complexity, for which there are several therapeutic modalities reported on scientific literature. Zygomatic implant placement is a viable option that features low morbidity and allows immediate prosthetic loading. The purpose of the present study was to determine the methodological quality of systematic reviews that assessed the effectiveness of zygomatic implants placed in atrophic maxillae.

**Material and Methods:**

Searches were conducted on Medline via Pubmed, LILACS, Dare Cochrane, Scopus, and Sigle via Open Grey up to June 2019.

**Results:**

Seven systematic reviews were eligible for Overview and comprised a total of 2313 patients, 4812 zygomatic implants, and a 96,72% success rate. Common surgical complications, in decreasing order, were: maxillary sinusitis, peri-implant mucositis, prosthetic fracture, and infections. Methodological quality was assessed using the AMSTAR 2 tool, which revealed that six systematic reviews showed critically low methodological quality and one review was assessed as of low methodological quality.

**Conclusions:**

Zygomatic implants seem to be an adequate option for atrophic maxilla rehabilitation, however, new studies with a higher methodological rigor are needed to provide more reliable results to professionals and patients undergoing this modality of oral rehabilitation.

** Key words:**Zygomatic Implants, dental Implants, complications, oral rehabilitation.

## Introduction

Oral rehabilitation with dental implants is a well addressed topic in Dentistry and is considered as the best alternative for the replacement of missing teeth that were lost by many reasons (1). However, a more careful planning is needed when an atrophic maxilla is rehabilitated, which represents a true challenge for implant dentistry (1,2).

Several techniques to rehabilitate atrophic maxillae are described in scientific literature, such as maxillary sinus augmentation, short implants, block grafting with intra and extra-oral donor sites, pterygoid implants, zygomatic implants, and many others (1-6). Each technique features advantages and disadvantages, and the surgeon must considerer the time involved on rehabilitation, the surgical morbidity, and the expected success rate to make the best choice for each individual case.

Maxillary reconstructions involve extensive bone grafting, such as maxillary sinus augmentation and iliac crest grafting, which demand a considerable amount of time for the final rehabilitation because an initial period of healing is need, which occurs in approximately 6 months (5,7). In addition, these interventions present a higher morbidity and may cause complications such as visible scarring, paresthesia, movement deficits, and infections (7,8).

The zygomatic implant is an alternative for maxillary reconstructions, in which an implant is placed into the body (i.e. the central portion) of the zygomatic bone, which has excellent quality in cortical bone density and provides the proper stability for immediate prosthetic loading. Altogether, final oral rehabilitation is facilitated and success rates are improved (9-12).

The zygomatic implant is usually placed bilaterally; nevertheless, a sufficient amount of bone in the anterior maxilla is needed so two regular implants can be placed in it to allow prosthetic stability. In patients with full maxillary atresia, whereas the placement of two anterior implants is not possible, two zygomatic implants can be placed bilaterally to obtain sufficient stability for prosthetic rehabilitation, including with immediate prosthetic loading and featuring high success rates (13-16).

Although the zygomatic implant is an excellent approach for atrophic maxillary treatment, its use also presents risks, such as maxillary sinusitis, oroantral fistula, infra-orbital paresthesia, peri-implant diseases, orbital perforations, and difficult in prosthetic adaptation, which may demand a more experienced and skilled surgeon to perform this treatment when compared to conventional implants (10-12).

The purpose of the present Overview was to answer the following focused question: what is the methodological quality of systematic reviews that assessed the effectiveness of zygomatic implants placed in atrophic maxillae?

## Material and Methods

A study protocol was developed a priori and was registered at the International Prospective Register of Systematic Reviews (PROSPERO – Protocol Registration ID: CRD42019121356). The present study was performed according to the PRISMA Statement (17).

- Search strategy

Online searches were conducted on Medline via PubMed [1966-2019], Lilacs [1982-2019], Dare Cochrane (up to 2019), Scopus [1996-2019], and Sigle via Open Grey [1980-2019]. The following search strategy was created at the MeSH platform and was inserted on the online databases: ("dental implants"[MeSH Terms] OR ("dental"[All Fields] AND "implants"[All Fields]) OR "dental implants"[All Fields]) AND zygomatic [All Fields] AND implants[All Fields]. A hand search was also performed to seek for relevant publications on the reference lists of included articles.

- Inclusion criteria.

1. Systematic review articles with or without meta-analysis that assessed the effectiveness of zygomatic implants;

2. Studies conducted with human patients, with no restriction of age, sex, or ancestrality;

3. Studies originally written in any language.

- Exclusion criteria:

1. Case reports, observational studies, randomized or non-randomized clinical trials, experimental studies, commentaries, expanded abstracts, and systematic review of systematic reviews (i.e. tertiary studies);

2. Studies that did not present data regarding the effectiveness of zygomatic implants;

3. Studies whose patients had one or many of the following: extensive dental caries, active periodontal disease, endodontic infections, diabetes, smoking habits, or other systemic diseases.

- Study selection

Study selection process was conducted independently by two researchers (PHHS and MWG), whom followed the same sequence of online databases to be searched, which was previously established by sortition. A third and more experienced reviewer (FJCL) (18-22) was consulted in cases of discrepancy in which a consensus could not be reached. Researchers followed eligibility criteria, excluded duplicated papers, and sought articles by titles and/or abstracts reading. Publications of relevance were fully read and then, eligible articles were included in the present Overview. This selection was performed up to June, 2019.

- Outcomes

Primary outcome was the methodological quality of systematic reviews regarding the effectiveness of zygomatic implants placed in atrophic maxillae. These data were expressed following the criteria of the AMSTAR 2 tool.

Secondary outcomes were: implant survival rate, bleeding index, and time of prosthetic loading (immediate, early, delayed, or non-reported), which were reported as percentages; marginal bone loss and peri-implant probing depth (expressed in millimeters); and complications related to the procedure, which were categorized by the type of complication and were expressed as percentages and absolute values.

- Methodological quality assessment

To determine the methodological quality level of included studies, the items of the AMSTAR 2 tool were used. This tool was used as reference standard so researchers could judge items and thus determine the level of quality of each one of included papers using a score that ranged from 0 (zero) to 16 (sixteen). The AMSTAR 2 tool considers items 2,4,7,9,11,13, and 15 as critical items, and the following answers were possible: yes, no, partially yes, or meta-analysis was not performed. Studies classified as of high methodological quality did not present negative answers in any critical item, and receive a negative answer in a maximum of one non-critical item. Studies that received negative answers in more than one non-critical item and no negative answers in critical items were classified as of moderate methodological quality. Studies with a negative answer in one critical item (with or without negative answers in non-critical items) were considered as of low methodological quality. Finally, studies which received negative answers in more than one critical item were considered as of critically low methodological quality.

- Statistical analysis

A narrative statistical approach was used for the primary outcome, i.e. the methodological quality of included studies. The aggregated data of secondary outcomes were described using quantitative descriptive statistics by means of weighted means and standard-deviations (if data were available on included studies). The Microsoft Excel 2010 was used for data processing and to obtain the weighted means and standard deviations.

## Results

**Figure 1:**
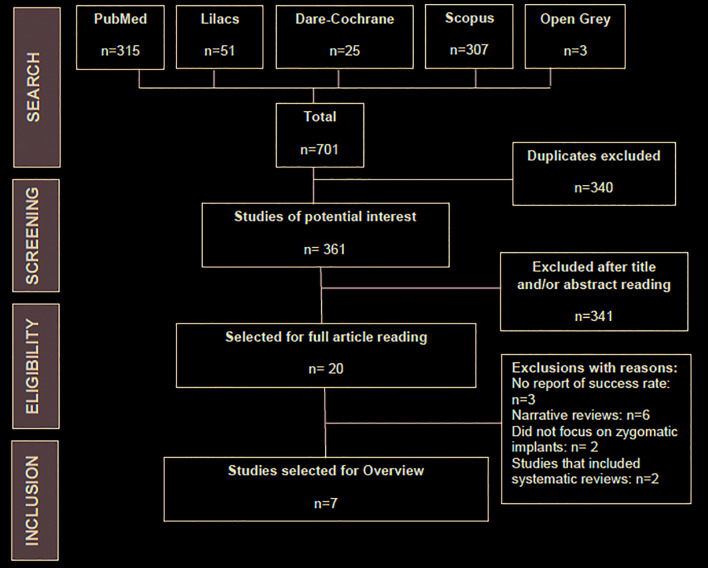
Flowchart of the study selection process.

Search yielded 701 results: 315 on Medline via PubMed, 51 on Lilacs, 25 on Dare Cochrane, 307 on Scopus, and 3 on Sigle via Open Grey. Duplicates were excluded (n=340) and 341 publications were excluded after title and/or abstract reading, totaling 681 initial exclusions. The 20 remaining articles were fully read and 13 more were excluded. The reasons for exclusions were: no report of implant success rate (n=3) (23-25); narrative reviews (n=6) (11,15,26-29); other rehabilitation techniques for atrophic maxillae, with no focus on zygomatic implants (n=2) (30,31); studies that included systematic reviews (n=2) (32,33). Finally, seven articles were eligible for Overview and underwent methodological quality assessment. The kappa index regarding the search and selection processes was of 89%, showing a strong agreement between reviewers. Fig. 1 shows details about the selection process.

- Quality assessment

Seven articles were selected for quality assessment using the AMSTAR 2 tool. Of which, only one paper (16) showed low methodological quality. The six others (10,34-38) presented critically low methodological quality. This assessment was performed by reviewers with an 83% kappa agreement level (strong agreement). Reviewers could not reach an agreement regarding the item 6 from AMSTAR 2 for one study (10) and the third reviewer (FJCL) was consulted to break the tie. Table 1 shows the results about methodological quality assessment.

**Table 1:**
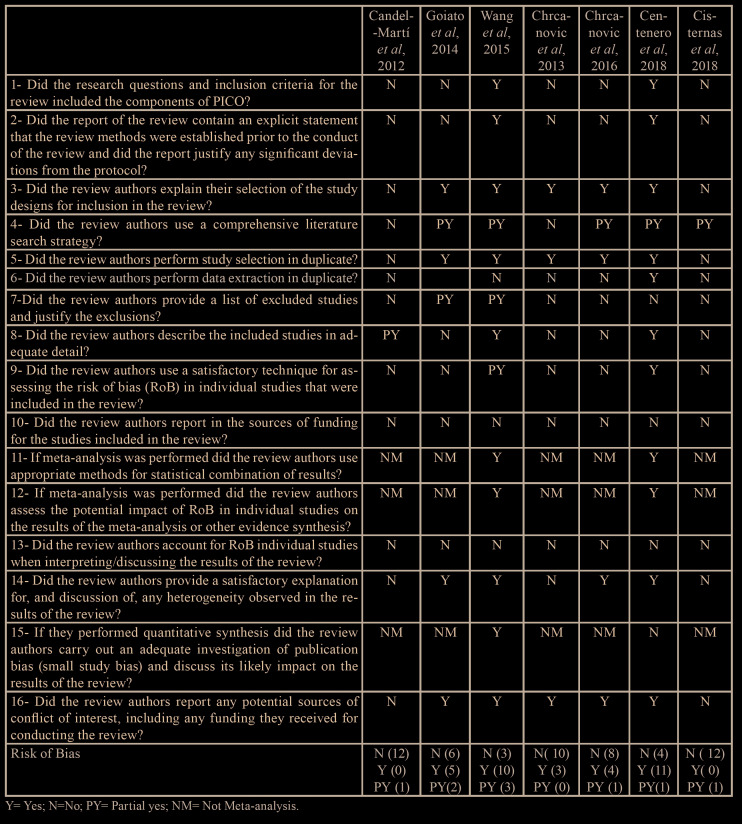
Quality assessment of systematic reviews using AMSTAR 2.

- Patients and implants

Systematic review articles that were included in the present Overview comprised a total of 185 primary studies, 5.440 patients, and 11.372 zygomatic implants (2.09 implants per patient). After the exclusion of repeated studies in different systematic reviews, the final numbers were of 73 different studies, 2.313 patients, and 4.812 zygomatic implants (2.08 implants per patient). The means of the minimum (10,16,34-38) and maximum (10,16,36,38) follow-up periods were of, respectively, 8 months and 26 days and 152 months and 17 days (global mean = 65 months). The survival rate of zygomatic implants was of 96.72% (standard deviation = 1.06). Bleeding index, marginal bone loss, and peri-implant probing depth were not reported in included studies. These results are shown on Table 2.

**Table 2:**
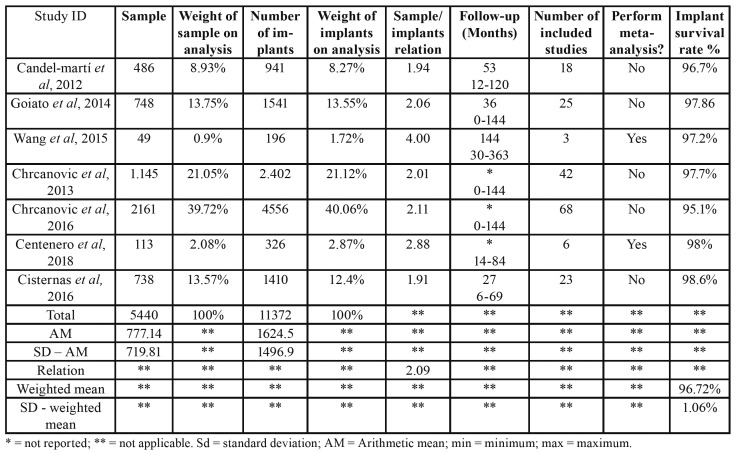
Patients, implants, follow-up period and survival rate of included studies.

- Prosthetic loading

Regarding the time for prosthetic loading, 43.35% implants received immediate or early loading and 29.69% implants received delayed loading. This outcome was not reported for 30.95% implants. When individual studies were analyzed, one paper (38) did not report this outcome; other publication (37) did not report this outcome for 745 implants, however, it reported that 2.219 received immediate loading and 1.592 implants received delayed loading; one study (34) reported only immediate or early loading and the remaining studies (10,16,35,37) provided data for immediate and delayed loading. Results from included systematic reviews are show on Table 3.

**Table 3:**
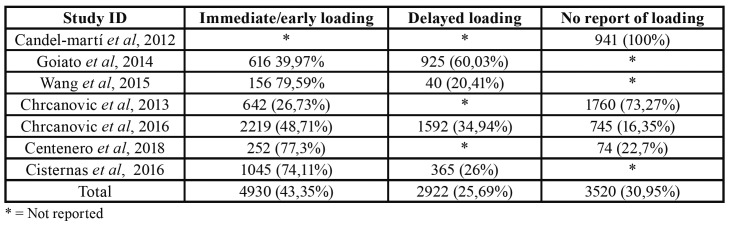
Prosthetic loading applied to zygomatic implants.

- Complications

Five studies reported complications (10,16,35-37). Maxillary sinusitis was the more frequent one and occurred in 128 cases, followed by peri-implant mucositis (75 cases), nerve injuries (34 cases), and oroantral fistula (30 cases). These numbers were obtained after the exclusion of duplicated articles; however, considering the weight that each study represented over the sample size, maxillary sinusitis showed weighted mean of 5.86%, followed by peri-implant mucositis (2.96%), prosthetic fracture (2.81%), infections (2.24%), nerve injuries (1.26%), oroantral fistula (1.20%), and other events such as hematoma/facial edema, labial lacerations, pain on the zygomatic region, deficient oral hygiene, and orbital perforation (1.33%, altogether). Two studies (34,36) did not report the occurrence of complications. Moreover, articles that accounted for complications using zygomatic implants, did not clarify if these complications lead to implant failure (10,16,35,37,38). The complications associated to the placement of zygomatic implants are described on Table 4 as absolute values and as percentages.

**Table 4:**
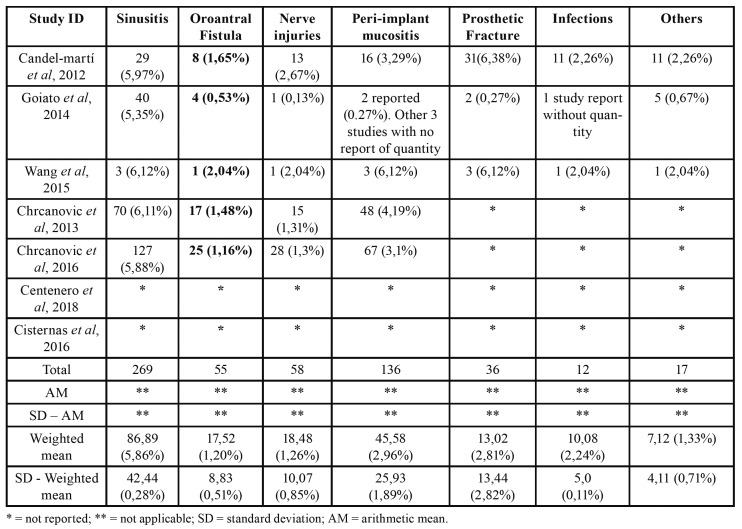
Complications reported in included studies.

## Discussion

The use of systematic reviews as worldwide reference standards for healthcare professionals is justified because this type of study is allocated at the top of scientific evidence pyramids. However, some studies show methodological flaws that reduce the overall quality and reliability of study results (20). This is clearly seen in the present study, which showed that eligible systematic reviews were assessed as of low and critically low methodological quality (10,34-38), according to the AMSTAR 2 criteria.

Many tools have been used to assess different aspects of systematic reviews, whereas AMSTAR is a reliable tool to determine the methodological quality of systematic reviews that included randomized clinical trials, only. A new tool (AMSTAR 2) was designed to assess the methodological quality of systematic reviews of randomized or non-randomized studies (including observational studies) and therefore was chosen for the present Overview (39).

Oral rehabilitation in atrophic maxillae require thorough planning and in many involves multiple surgical procedures, which causes considerable morbidity. Zygomatic implants aroused as a viable option for these cases, since bone grafting is not necessary, it features excellent stability, and allows immediate prosthetic loading with high success rates (circa 97%) (14-16). The present study showed aggregated implant success rate of 96,72%, which corroborate with these studies.

This is particularly important since the effectiveness of zygomatic implants is similar to the effectiveness of standard-sized dental implants placed after maxillary sinus augmentation, which needs bone grafting and demands a higher rehabilitation period (5), and of short implants(<6mm) (40), which although their effectiveness for atrophic maxillae, a residual bone quantity is needed for implant placement and usually is not possible to perform immediate loading (40,41).

Although dental implant placement presents high survival rates, several important complications should be taken into account, such as maxillary sinusitis, peri-implant mucositis, prosthetic fractures, nerve injuries, and oroantral fistula (32,33). Maxillary sinusitis is the most common one and corroborates with data from the present study.

It is worth mentioning that included systematic reviews did not report (when they report it) if one or more of the aforementioned complications where the cause for zygomatic implant failure. New technologies have been used to reduce the occurrence of such complications and to increase the predictability and precision of the procedure. Among these technologies, the use of guided surgeries, prototyping biomodels, and bone measures in specific software should be mentioned (42,43).

It is also known that complications are more likely to occur depending on the surgical technique (e.g. if intra 9 or extra-sinus 44), which also influences the prosthetic positioning (45). Still, only two out of seven included articles reported the type of surgical technique used to place the zygomatic implants (10,38). Complications may also occur in other modalities of oral rehabilitations, showing similar to the ones that occur with zygomatic implants, such as maxillary sinusitis, maxillary sinus perforations, and peri-implant mucositis (5,41).

One must highlight that five out of seven included studies did not perform meta-analysis and did not had a control group (10,35-38). In addition, some studies presented unclear inclusion criteria (5,38), which favored the inclusion of primary studies with low level of scientific evidence. These data are available on AMSTAR 2 assessment Table, in which two studies did not receive any positive score (36,38), showing how low was the methodological quality of obtained results and, consequently, of the primary studies included on eligible systematic reviews.

The registration of an a priori review protocol, although is not a mandatory item, is highly recommended and is a critical item on AMSTAR 2; still, only two studies reported the registration of the review protocol (16,34). The present overview, and other reviews of the involved research, was registered on PROSPERO, which reduces the study risk of bias.

It is important to emphasize that the zygomatic implants evaluated in this study, were evaluated through their survival rate and not exactly in their success rate, a term that could generate a misinterpretation of the results since the studies selected in this overview did not evaluate peri-implant bone loss or bleeding index, important factors that could indicate peri-implantitis, which could affect the success rate of zygomatic implants.

Zygomatic implants placed in atrophic maxilla showed high survival rate and few complications. Included systematic reviews showed low methodological quality, which reduced the scientific evidence level. New studies with higher methodological rigor and with the inclusion of outcomes that may predict implant failure, such as bleeding index, probing depth, and peri-implant bone loss, are necessary to provided precise data that may aid dental surgeons in the proper planning and placement of zygomatic implants.
